# The Relation between Urinary Tract Infection and Febrile Seizure

**Published:** 2018

**Authors:** Abolfazl MAHYAR, Parviz AYAZI, Elaheh AZIMI, Reza DALIRANI, Ameneh BARIKANI, Shiva ESMAEILY

**Affiliations:** 1 Qazvin Children Hospital, Qazvin University of Medical Sciences, Qazvin, Iran; 2 Mofid Children Hospital, Pediatric Nephrology Department, Shahid Beheshti University of Medical Sciences, Tehran, Iran

**Keywords:** Febrile seizure, Urinary tract infection, Children

## Abstract

**Objectives:**

Febrile seizure is the most common type of seizure among children. Identification of factors involved in febrile seizure is highly critical. The present study was conducted to determine the association between children’s urinary tract infection and febrile seizure.

**Materials & Methods:**

In this case-control study, 165 children with simple febrile seizure (case group) were compared with 165 children with fever and without seizure (control group) in terms of urinary tract infection (UTI) in Qazvin, central Iran in 2015-2016. The age of children was between 6 months to 5 yr.

**Results:**

Among 165 children with febrile seizure, 25 (15.2%) had urinary tract infection. In the control group, only 2 patients (1.2%) had UTI. There was significant difference between two groups regarding urinary tract infection (*P*=0.001). Among 25 children with UTI in the case group, 17 children (68%) had acute pyelonephritis, and the remaining 8 children (32%) had cystitis. The two patients with UTI in control group had cystitis (*P*=0.055).

**Conclusion:**

Urinary tract infection could be a risk factor for febrile seizure. Therefore, all patients with febrile seizure are examined in terms of urinary tract infection.

## Introduction

Febrile seizure is the most common type of seizure among children. Febrile seizure refers to a type of seizure which occurs if temperature of child’s fever is equal to or higher than 38 °C. These patients have not central nervous system infection (such as meningitis, encephalitis, etc.), metabolic disease and afebrile seizure. The disease is usually observed among children within age range of 6 to 60 months. The age peak of febrile seizure is 18 months old. 

The prevalence of febrile seizure ranges from 2% to 5% (1-4). Febrile seizure is divided into simple and complex types. Regarding the simple type, seizures are usually of tonic-clonic type. The seizures do occur once a day and take less than 15 min. Concerning complex type of seizure, it is usually focal and occurs more than once a day and each seizure takes more than 15 min (1-2). Febrile seizure usually has a good prognostic outcome, but it may be lead to epilepsy in 2%-7% of the cases (1). 

Although the etiology of febrile seizure is imprecise, some reports point to the role of certain factors such as genetics, infections and deficiency of certain trace elements (5-7). Considering the role of some viral infections in febrile seizure (6), the question is raised for us that “Do bacterial infections such as UTI could provide the conditions for febrile seizure?” Urinary tract infection (UTI) is one of the common diseases of children. In 75%-90% of the cases, *Escherichia* *coli* is the agent. The prevalence of UTI among females and males are 3%-5% and 1%, respectively (8, 9). Previous studies on the association between UTI and febrile seizure offer contradictory results (9-12). 

Considering the high prevalence of febrile seizure and significance of identification of influential factors, the present study was conducted. 

## Materials & Methods

In this case-control study, 165 children with simple febrile seizure (case group) were compared with 165 children with fever and without seizure (control group) in terms of UTI. The study was conducted in Qazvin Children Hospital, Qazvin University of Medical Sciences, Qazvin, central Iran in 2015-2016. In both groups, children’s age ranged from 6 months to 5 year. Sample size was calculated as: =α0.05 1-α=0.95 β=20% 1-β (power)=80%, P_1_=1 (Hypothetical proportion of control with exposure), P_2_=6.6 (Hypothetical proportion of cases with exposure), OR=7 (least extreme odds ratio to be detected) and following formula: 

$n=\frac{2{\left(Z_{1-\frac{\alpha }{2}}+Z_{1-\beta }\right)}^{2}\times \bar{P}(1-\bar{P})}{{\left(\mathrm{p1}-\mathrm{p2}\right)}^{2}}$

Sampling was frequently done until intended sample size was achieved. Febrile seizures were defined seizures that occur between the age of 6 and 60 months with a temperature of 38 °C or higher, that are not the result of central nervous system infection or any metabolic imbalance, and that occur in the absence of a history of prior afebrile seizures. A simple febrile seizure was defined a primary generalized, usually tonic-clonic, attack associated with fever, lasting for a maximum of 15 min, and not recurrent within a 24-h period (1-4). The children with complex febrile seizure, epilepsy, developmental delay, abnormal neurologic examinations, central nervous system infections (e.g. Meningitis), electrolyte disorders (e.g. hypocalcemia and hyponatremia), metabolic diseases (e.g. Phenylketonuria) and any factor that justifies seizure as well as uncircumcised children were excluded from the study. 

**Table 1:** Comparison of variables between case and control group

Variables	Case group	Control group	*P-*value
	n=165	n= 165	
Sex (male/female) 1	97/68	92/73	0.65
Age (month) 2	14±(12)	12±(16.50	0.55
Weight (kg) 2	11±(4)	11±6	0.79
Height (cm) 2	76±(10)	75±(15)	0.54
Head circumference (cm) 2	48±(4)	48±(3)	0.83
Temperature (^0^C)	38.4±0.8	38.3±0.9	0.2
Hemoglobin (gr/dl)	11.3 ±1.4	11.2±1.5	0.85

1 Chi-square test,

2 Median±IQR (Mann–Whitney U test),

3 Mean±SD (*t*-test)

**Table 2:** Comparison of etiologic causes of febrile seizure in case and control group

Type of disease	Case group	Control group	*P*-value
	n (%)	n(%)	
Acute gastroenteritis	77(46.7)	80(48.5)	0.56
Upper respiratory infection	55(33.3)	60(36.4)	0.92
Pneumonia	6(3.6)	13(7.9)	0.59
Acute otitis media	2(1.2)	10(6)	0.02
UTI	25(15.2)	2(1.2)	0.001
Total	165(100%)	165(100%)	

Chi-square test

**Table 3:** Comparison of UTI in case and control groups

UTI	Case group	Control group	*P*-value
Yes	25(15.2)	2(1.2)	0.001
No	140(84.8)	163(98.8)	
Total	165(100)	165(100)	

Chi-square test

The control group was selected by group matching from the children with fever and without seizure visited the hospital due to common diseases such as infection of upper respiratory tract and diarrhea. Both groups were local and residents of Qazvin. All male members of both groups had been circumcised. UTI was defined as: positive urine culture (urine culture positive for more than 10^5^ CFU/mL of a single pathogen in a midstream urine sample or clean catch method or 10^4^ CFU/mL of a single pathogen via urinary catheterization, or presence of any number of colonies of an organism in urine culture taken by suprapubic method (13). 

The project was approved by the Ethics Committee of Qazvin University of Medical Sciences (project no: 1121). Then children’s parents were briefed on the study objectives and methods. After written parental consent was obtained for the children’s participation in the study and verbal consent was obtained from the older children.

The demographic information (e.g. age, sex, height, weight, and perimeter of head), clinical symptoms and laboratory findings (e.g. hemoglobin, type of UTI, and microorganism) of patients were collected and registered. Measurement of height, weight, perimeter of head and temperature of patients was done through available standard methods (14). All of the intended tests were conducted in the laboratory of Qazvin Children Hospital. 

The data were analyzed through Chi-square test and *t*-test. To conduct intended analyses, SPSS Software (ver. 20, Chicago, IL, USA) was used. *P*-value of less than 0.05 was considered significant. 

## Results

Among 165 children in case group, 97 patients were male (58.8%).‌ In the control group, 92 patients were male (55.8%) (*P*=0.65). No significant differences were found between the two groups in terms of age, sex, height, circumference of head, temperature and hemoglobin concentration (Table 1). Among 165 children with febrile seizure, 25 patients (15.2%) had UTI. In the control group, 2 patients (1.2%) had UTI (*P*=0.001) (Tables 2 and 3). There was significant difference between two groups regarding UTI (*P*=0.001). Among 25 children with UTI belonging to case group, 17 patients (68%) had acute pyelonephritis, and 8 patients (32%) had cystitis. In the control group, two patients with UTI had cystitis (*P*=0.055).

The most common organism causing UTI in children was *E. coli*. The most sensitivity was seen to amikacin, gentamicin, ceftriaxone and nalidixic acid, and the highest drug resistance was found to ampicillin, cefalexin, and *trimethoprim-sulfamethoxazole*. 

## Discussion

The findings of present study suggested that the prevalence of UTI in children with simple febrile seizure was higher than control group. The findings of studies on the role of UTIs in febrile seizure are contradictory (9-12, 15, 16). A study was conducted on 137 children with simple and complex febrile seizure with age range of 1 month to 5 year. The prevalence of UTI in children with febrile seizure was 6.6%. The author's recommended urinalysis and urine culture test for all patients with febrile seizure so that probable UTI cases could be identified (9). In another study conducted on 228 children with febrile seizure in the age range of 1-71 months (mean age: 24 months), 5%-12% of children had bacteriuria. The authors emphasized the significance of UTI diagnostic tests for children with febrile seizure (10).

In contrast to the above-mentioned studies, other studies pointed to insignificant difference between children with and without febrile seizure in terms of prevalence of UTI (11, 12, 15, 16). In a study on 243 children with febrile seizure (mean range: 1.9±-0.96), the prevalence of UTI in such children was 0.7% (11). A similar study was conducted on 171 children with simple febrile seizure (mean age: 21 months) and pointed to 5.9% prevalence of UTI. The prevalence of UTI was reported as 2.6% (15). Among children aged less than 2 yr diagnosed with UTI; only 2 patients (2.6%) had febrile seizure (16). In all of the above-mentioned studies, control group was lacking and the obtained results were compared with mean prevalence of UTI in the public. In the present case-control study, the prevalence of UTI in children with febrile seizure was 15.2%. In addition, a significant difference was found between the group with febrile seizure and the group with fever but without seizure in terms of UTI. The findings of present study are supported by results of two studies (9, 10). Difference in the results of the above-mentioned studies might be due to variable factors such as age of patients, size of sample, type of study, method of sampling and circumcision of male patients. 

Based on the our results, diagnosis of UTI in children with febrile seizure is significant due to following reasons: 1- Clinical manifestations of UTI during breastfeeding and early childhood is often accompanied by fever and it rarely has local symptoms such as pain, tenderness of flank, stomachache, as well as explicit urinary symptoms (17). 2- Usually the cause of fever in these patients is viral and is not usually treated with antibiotics. As a resultif the febrile patient has UTI, it will not be detected (6). Lack of quick diagnosis and treatment of UTI, especially acute pyelonephritis, could lead to dangerous consequences such as kidney scarring, hypertension and chronic kidney failure (8.^17^). Probability of kidney scarring in low-age children is higher than older children. In addition, patients with UTI may be inflicted with urinary system abnormalities such as reflux and hydronephrosis. These abnormalities might increase the probability of recurrence of UTI and also add to likelihood of kidney scarring (17).

Among limitations of present study we can point to the following cases: 1) Conducting the study solely in one center; 2) Lack of study on children with complex febrile seizure. The authors recommend further studies in this regard: such as multi-center study, study on complex febrile seizures and study on pathophysiology of this association. 

**In conclusion, **considering the high prevalence of UTI (15.2%), we recommend the urinalysis and urine culture test for all children with febrile seizure, so that UTI cases could be identified. 
